# A Comparison of the Results of Two Different Double-Row Repair Techniques in Arthroscopic Repair of Rotator Cuff Tears

**DOI:** 10.3390/medicina61040674

**Published:** 2025-04-06

**Authors:** Gökhan Ünlü, Mehmet Faruk Çatma, Ahmet Burak Satılmış, Tolgahan Cengiz, Serhan Ünlü, Mustafa Erdem, Önder Ersan

**Affiliations:** 1 Department of Orthopedics and Traumatology, Medicalpark Gebze Hospital, 41400 Kocaeli, Turkey; 2 Department of Orthopedics and Traumatology, Etlik City Hospital, 06010 Ankara, Turkey; farukcatma@hotmail.com (M.F.Ç.); onersan@gmail.com (Ö.E.); 3 Department of Orthopedics and Traumatology, Taşköprü State Hospital, 37400 Kastamonu, Turkey; absatilmis@gmail.com (A.B.S.); tolgahancengiz@hotmail.com (T.C.); 4 Department of Orthopedics and Traumatology, Medicalpark Keçiören Hospital, 06010 Ankara, Turkey; serhanunlu@yahoo.com; 5 Department of Orthopedics and Traumatology, Afyonkarahisar State Hospital, 03030 Afyon, Turkey; drerdem34@gmail.com

**Keywords:** shoulder injuries, rotator cuff injuries, double-row repair techniques, arthroscopic repair

## Abstract

*Background and Objectives*: Shoulder pain, mainly involving rotator cuff tears, is a common type of musculoskeletal pain that significantly impairs quality of life. Arthroscopic rotator cuff repair has become the gold standard for treating symptomatic, full-thickness rotator cuff tears. Double-row repair techniques are widely used because of their superior fixation and healing results. However, fewer implants may reduce treatment costs and raise questions about the impact on clinical outcomes and re-tear rates. This study compares the functional outcomes and re-tear rates of two transosseous-like double-row repair techniques: one anchor and one push lock (Group 1), and two anchors and two push locks (Group 2). *Materials and Methods*: A prospective, randomized, single-blind study was conducted on 53 patients undergoing arthroscopic repair for crescent-shaped rotator cuff tears (3–5 cm). Before surgery and 24 months after surgery, patients were evaluated for shoulder function using Constant–Murley scores and shoulder abduction angles. MRI was used to assess re-tear rates. *Results*: Both groups showed significant postoperative improvement in Constant scores (Group 1: 84.1; Group 2: 84.0; *p* > 0.05). Re-tear rates were slightly higher in Group 1 (23.1%) than in Group 2 (18.5%), but this was not statistically significant (*p* > 0.05). Shoulder abduction angles improved similarly between groups, with no significant difference in outcome. Despite higher costs and longer operative times, the two-anchor technique provided more stable fixation, but its functional outcomes were comparable to the single-anchor method. *Conclusions*: Using fewer implants in a double-row repair provides comparable functional outcomes and re-tear rates, and offers surgeons a cost-effective alternative, especially at the beginning of their learning curve. However, the two-anchor technique may be more beneficial in cases requiring improved mechanical stability. These findings provide valuable information to balance cost and effectiveness in rotator cuff repair.

## 1. Introduction

The third most common cause of pain originating from the musculoskeletal system, after lower back and knee joint pain, is shoulder pain. The shoulder joint is the most mobile joint in the body. A large part of the joint’s function is provided by the rotator cuff (RC), and pathologies of this muscle group negatively affect quality of life. Rotator cuff tears are found in approximately 20% of the population over 60 years of age, and are a major contributor to shoulder pain and dysfunction in the elderly [1]. Moulton et al. first described rotator cuff rupture and described its epidemiology, pathophysiology, and natural history [2]. Petri et al. stated nonsurgical treatment options [3]. These are still used as first-line treatment options. Hawi et al. described debridement procedures in rotator cuff tears [4]. Rotator cuff tear repair was described by Spiegel et al., and the advantages of single-row repair were emphasized [5]. Mook et al. and Greenspoon et al. described double-row repair with knotless connections [6]. Patients with symptomatic full-thickness rotator cuff tears can be treated arthroscopically, and significant functional improvement is seen in these patients [5]. Arthroscopic instrumentation and surgical techniques have rapidly developed recently, and arthroscopic RC repair has become more popular. Postoperative rehabilitation plays a crucial role in functional recovery after rotator cuff repair. Recent evidence suggests that early rehabilitation protocols may offer advantages over traditional approaches regarding short-term range of motion outcomes and strength improvement [7].

The double-row repair technique is the most up-to-date and widely used approach in the arthroscopic treatment of rotator cuff tears. It has been reported that the rotator cuff attachment site footprint cannot be restored by the single-row repair method, and failure rates are higher [8,9]. This was the precursor to the development of double-row repair, which is a more reliable fixation. The double-row repair method provides better footprint restoration and increases the contact area for healing. In this way, the contact area of the bone surface of the torn cuff is increased, and a water-impermeable repair is obtained.

Several studies have shown that patients with double-row repairs have the lowest re-rupture rate after repair [10]. Double-row repair can be applied with several methods and implants. In the method described by Lo and Burkhart, two implants are used, one medial and one lateral [11]. In another method defined by Mook and Greenspoon, four implants are used, two medial and two lateral [6]. In light of this information, this study aimed to report prospective comparative results on whether the same success could be achieved by repairing tears of the same type and size in a shorter time by using fewer implants. In addition to clinical outcomes, this study also emphasizes the cost-effectiveness of the techniques—especially the use of fewer implants—considering their relevance in resource-limited settings.

## 2. Materials and Methods

This prospective, randomized, single-blind study was initially designed with 78 patients diagnosed with symptomatic rotator cuff tears between 2014 and 2015 who did not adequately respond to conservative treatment and were scheduled to undergo surgery using two different double-row techniques. The inclusion criteria for the study were a 3–5 cm crescent-shaped tear, failure to respond to conservative treatment, and suitability for surgery; the exclusion criteria were partial tears, irreparable tears, Goutallier stage 4 fatty degeneration, L-shaped and large tears, and patients who did not complete the 2-year follow-up. Initially, 78 patients were included; 25 were excluded during surgery or follow-up due to these criteria.

Patients were randomized using a computer-generated random number list, and group allocations were sealed in opaque envelopes opened on the day of surgery. A single-blind design was used, where an independent, blinded orthopedic specialist performed all clinical evaluations. The first group underwent surgery using one anchor and one push lock (Group 1), while the second group underwent surgery using two anchors and two push locks (Group 2). Accordingly, one patient in Group 1 was excluded, as they had large tears (3–5 cm) according to the DeOrio and Cofield classification. One patient experienced implant-related complications during surgery, and another patient’s tear was evaluated as a massive and irreparable tear. Seven patients did not attend follow-up and were excluded from the study. In total, 10 patients were excluded from the evaluation, and 26 were included in Group 1. In Group 2, three patients were excluded as they had large tears (3–5 cm) according to the DeOrio and Cofield classification. One patient was excluded because the tear was classified as an L-shaped tear, according to Ellman and Gartsman. Eleven patients did not attend follow-up and were excluded from the study. In total, 15 patients were excluded from the evaluation, and 27 were included in Group 2.

An orthopedist not involved in the study evaluated the patients in both groups regarding age, gender, dominant side, preoperative and postoperative abduction angles, and Constant and Murley scores. Preoperatively, anteroposterior (AP) radiographs and magnetic resonance imaging (MRI) were taken of all the patients as standard. The diagnosis of rotator cuff tear was confirmed on preoperative MRI and in a physical examination through range of movement (ROM) evaluation.

### 2.1. Surgical Procedure

A single surgeon performed the operation with the patient in the beach-chair position under general anesthesia. Posterior, anterior, and lateral portals were used. Intra-articular pathologies were determined and treated. The tear size was measured using the arthroscopic probe in the sagittal plane. First, subacromial decompression was completed. In cases where >50% of the biceps tendon was torn, tenotomy or tenodesis was performed, provided that the patient’s age was 55 years or over [12]. In the cuff repair of both patient groups, anchors and push locks of the same make (Arthrex, Naples, FL, USA) were used with the Samsung Medical Centre (SMC) sliding locking knot technique.

**Figure 1:**
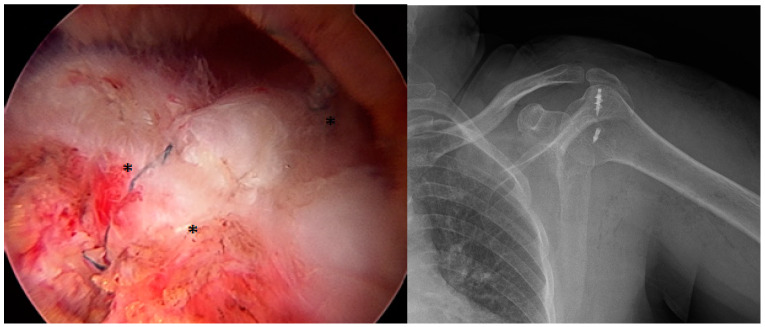
The arthroscopic appearance of a rotator cuff repair made with one anchor and one push lock. * Two threads of different colors fixed with a push lock and knotted over the cuff.

In Group 1 patients, one anchor and one push lock were used. First, one anchor with four threads was screwed at an angle of 45° to the lateral joint cartilage, to be in the center of the tear. Then, using a Scorpion suture passer (Arthrex, Naples, FL, USA), the first thread was taken from the anterior corner of the tear to the anterior portal. Then, the other leg of the same thread and the first leg of the other thread were passed. The second and third threads that were passed were knotted in the portal. The knot was seated over the cuff by pulling the free ends. The final leg of the second thread was passed from the most posterior end of the cuff. The two different colored threads remaining free were fixed to be more lateral to the anchor and tightened with a push lock. The cuff repair was completed by cutting the free threads (Figure 1).

**Figure 2:**
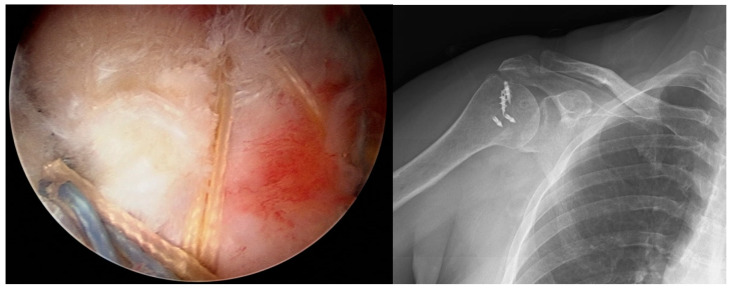
Arthroscopic appearance of rotator cuff repair made with two anchors and two push locks.

In Group 2 patients, two anchors and two push locks were used. First, one anchor with two threads was screwed to the footprint prepared in the humerus, to be in the anterior third of the width of the tear. Starting from the anterior, two legs of the same thread were passed through the cuff using the Scorpion suture passer. The cuff was positioned over the anchor using the SMC sliding, locking, and knot technique. The thread ends were carried to the anterior portal. The same procedure was applied for the two legs of the other thread and the threads of the other anchor, from anterior to posterior, respectively. Thus, 4 knots formed from 8 threads were obtained over the cuff. Keeping the threads of the first and third knots tight, the distal of the first anchor was tapped with the push lock, the threads of the second and fourth knots were held tight, and the distal of the second anchor was tapped with the other push lock. The free threads were cut, and the cuff repair was completed in a water-impermeable manner (Figure 2).

### 2.2. Clinical Evaluation

Shoulder functions were evaluated as per the Constant–Murley grading system preoperatively and in the 24th month postoperatively. The total Constant score was classified under four headings: excellent (90–100), good (80–89), moderate (70–79), and poor (<70). The patients in both groups were evaluated preoperatively and postoperatively regarding shoulder abduction angles. In the final follow-up examinations, MRI was taken of cases with re-rupture suspected based on the physical examination and of those with a poor Constant score. The patients’ re-rupture rates and functional results were compared between the two groups.

### 2.3. Rehabilitation Protocol

Postoperatively, an arm sling with a belt and abduction cushion was applied to the patients with the arm in 45° abduction, and was worn for 4–6 weeks. On postoperative day 1, phase 1 exercises were started, involving elbow and wrist exercises. From the 3rd week onwards, pendular shoulder exercises were added, and from the 6th week, phase 2–3 active range of movement exercises were started under the guidance of a physiotherapist.

### 2.4. Statistical Analysis

The data obtained in the study were analyzed statistically using SPSS for Windows vn. 20.00 software (IBM Inc., Armonk, NY, USA). Continuous variables, such as patient age, duration of complaint, and Constant score, were stated as mean ± standard deviation (SD) values. Categorical variables, such as gender, affected side, and dominant side, were stated as number (n) and percentage (%). Conformity of the data to normal distribution was assessed with the Kolmogorov–Smirnov test, and data not showing normal distribution were compared using the Mann–Whitney U-test and the Pearson test. A value of *p* < 0.05 was accepted as statistically significant.

Ethics Committee Approval: The Local Ethics Committee approved this randomized controlled trial (date: 24 July 2017, decision no: 40/06).

## 3. Results

Patients with a crescent tear 3–5 cm in size were grouped according to the two different suturing techniques used in the repair. Group 1 comprised 26 patients with a tear repaired with one anchor and one push lock, and Group 2 comprised 27 patients with a tear repaired with two anchors and two push locks. Group 1 comprised 18 (69.2%) females and 8 (30.8%) males, with a mean age of 59.07. Group 2 comprised 22 (81.4%) females and 5 (18.6%) males, with a mean age of 62 years (Table 1). No statistically significant differences were determined between the groups regarding age or gender (*p* > 0.05).

Pathology was determined in the dominant extremity in 19 patients in Group 1 and 17 in Group 2. The operation was performed on the non-dominant side in 16 patients, and in both extremities in 1 patient. No statistically significant difference was determined between the groups concerning the dominant extremity status (*p* > 0.05).

In Group 1, two patients underwent Bankart repair in glenohumeral arthroscopy, and two underwent SLAP lesion repair. No additional pathology was determined in the other patients. In Group 2, two patients underwent Bankart repair, one underwent SLAP lesion repair, one underwent biceps tenodesis, one underwent biceps tenotomy, and one underwent subscapularis tendon repair. No additional pathology was determined in the other patients. Acromioplasty was applied to all the patients (Table 1).

The patients in both groups were evaluated preoperatively and postoperatively regarding shoulder abduction angles. No statistically significant difference was determined between the two groups concerning the preoperative (*p* = 0.15) and postoperative (*p* = 0.587) shoulder abduction angles (Table 2). The postoperative shoulder abduction angle was 151–180° in 15 (57.7%) patients in Group 1 and 19 (70.4%) patients in Group 2. No statistically significant difference was determined between Group 1 and Group 2 concerning the preoperative (*p* = 0.586) and postoperative (*p* = 943) Constant scores (Table 3). When Groups 1 and 2 were compared in terms of Constant score as excellent–good–moderate, no statistically significant difference was found between the groups (*p* = 0.744) (Table 4). Postoperative MRI revealed a re-tear rate of 23.1% in Group 1 and 18.5% in Group 2, and no statistically significant difference was found between the groups (*p* = 0.682). All patients underwent standard preoperative AP radiographs and MRI. Shoulder range of motion was assessed using abduction angles, which are presented in Table 2 and Table 3.

## 4. Discussion

Arthroscopic techniques have become the standard of care in managing full-thickness rotator cuff tears, yielding favorable functional results [8]. Several studies of arthroscopic treatment of rotator cuff tears have reported satisfactory results regarding pain and shoulder function [13,14]. In addition, imaging studies in the literature have reported re-rupture rates between 29% and 94%. The re-rupture rate is higher, especially in older patients with a massive tear [15,16]. In the current study, 53 patients with a complete-layer rotator cuff tear who had not sufficiently responded to conservative treatment received arthroscopic rotator cuff repair.

Several surgical methods for treating rotator cuff tears have been compared in the literature. Open, mini-open, and arthroscopic repairs have been the subject of comparisons, as well as several techniques, such as single-row and double-row repair, transosseous repair, and transosseous-like repair, which are determined according to the differences in the number of anchors and suture passes used. In the current study, two different methods were compared: both transosseous-like techniques, but using different anchors.

The need to use fewer implants when using a transosseous-like technique can be due to restrictions on treatment costs in different centers. The bone–tendon interface formed with fewer implants will undoubtedly be smaller. Biological healing must be supported by mechanical stability. Recent studies have shown transosseous-equivalent repairs to provide the best strength and footprint pressure [17,18]. This pressure and strength is vital for healing between the tendon and bone. It has been reported in recent studies that tendon healing is provided by vascular growth progressing from the bleeding bone bed [19,20]. Strong healing is obtained when the area of the footprint surface in contact with the tendon increases.

Based on the available data in the literature, the current study’s hypothesis was formed: that the shoulder functional results would be insufficient, and the re-rupture rate would be higher in Group 1, where the operation was performed using fewer implants. In examining the data of the patients in the current study, no significant difference was found between the two groups concerning the shoulder functional scores. This finding is consistent with a study by Nicholas et al., in which single-row and double-row repairs were compared [21]. In a 2015 study by Wang et al., in the results of 102 patients in a double-row repair group, the Constant score was found to be at a mean of 84.2 at 2 years postoperatively [22]. The Constant scores in the current study were at a mean of 84.08 in Group 1 and 84.0 in Group 2.

Some studies have reported that pain and clinical results are unaffected in patients who develop re-rupture [23,24], and some authors have reported lower clinical results [25,26]. These different results could be related to various patient populations, sample sizes, and measurement techniques in multiple studies. In a meta-analysis by Yang et al., which collated the data of 29 studies, there was a decrease in Constant, UCLA, and ASES scores, pain, and abduction strength in patients who developed re-rupture. However, no statistically significant difference was determined between the repair techniques. It was concluded that re-rupture had no significant effect on pain, but did affect the postoperative clinical results [27]. Similarly, in the current study, although an increase was observed in the Constant scores of patients with re-rupture in both groups compared to the preoperative values, this was determined to be lower than that of the patients with an intact rotator cuff. These findings support previous studies that have shown that the clinical outcome is affected by re-rupture.

Yamamoto et al. reported that the dominant extremity was affected in 59% of patients in a group with rotator cuff tears [28]. Wang et al. found that the right shoulder was affected at a rate of 52.9% in a patient group treated with double-row repair [22]. In a 2016 meta-analysis by Yang et al. involving 1391 patients, 81 dominant extremities were affected in 76.3% of patients with double-row repair [27]. In the current study, the right side was affected in 67.9%, and the dominant extremity was affected in 69.8% of the total 53 patients in both groups. No statistically significant difference was determined between the two groups concerning the affected and dominant extremity.

Subacromial decompression during rotator cuff repair aims to increase the supraspinatus exit volume by obtaining a smooth surface below the acromion and acromioclavicular joint. This eliminates impingement and prevents cuff tears, which could form again with extrinsic mechanisms [29]. However, whether arthroscopic subacromial decompression should be performed routinely as a component of rotator cuff repair surgery remains a matter of debate [30]. In a randomized, prospective study by Milano et al., 80 patients were evaluated following rotator cuff repair, half of whom had arthroscopic subacromial decompression and half of whom did not. In the early follow-up results, the functional results were reported to be the same in both groups, and it was suggested that subacromial decompression does not affect functional results [31]. However, the follow-up period was short in that study, and high success rates in studies reporting long-term follow-up results have revealed the need for arthroscopic subacromial decompression in rotator cuff surgery [30,32,33]. Voloshin et al. reported that during rotator cuff tear surgery, the subacromial bursa, where catabolic enzymes such as matrix metalloproteinases, cytokines, and cyclooxygenases are released, must be cleaned to prevent degeneration of the rotator cuff [34]. In another study by Randelli et al., local growth factors were expressed in the region following acromioplasty, increasing potential healing in the rotator cuff [35]. Considering the effect of increasing the healing potential in the rotator cuff, acromioplasty was performed regardless of acromion type in all the current study patients who developed rotator cuff tears associated with a degenerative process. Bursectomy was applied to all the patients to prevent degeneration and ensure a comfortable repair procedure. All the osteophytes in the acromioclavicular joint patients were cleared to avoid causing re-rupture.

Despite similar functional outcomes observed in both groups, the single-anchor technique demonstrated superior cost-effectiveness, due to reduced implant use and shorter surgical time. This may offer a distinct advantage for surgeons in the early stages of their learning curve or for healthcare systems operating under financial constraints. Furthermore, emerging artificial intelligence (AI) developments have opened new avenues in optimizing postoperative care. Machine learning and deep learning technologies have shown promise in tailoring rehabilitation strategies and predicting re-tear risks based on patient-specific data, potentially enhancing long-term outcomes in rotator cuff repair [36,37].

The greatest strength of this study is that it is one of the rare prospective randomized trials that directly compare the functional outcomes and re-tear rates of rotator cuff repair using single- or double-anchor transosseous-like techniques. The findings provide critical guidance for surgeons, particularly those early in their learning curve or in resource-limited settings, to balance cost-effectiveness with fixation stability. However, this study also has certain limitations. The follow-up period of 24 months was insufficient to state long-term results. The examination of these results is ongoing, as there is a need for studies with longer-term results. A second limitation was that a single evaluator examined the MRI scans, and there could have been false negative or positive results because of implant artifacts. Another limitation is that only patients with a specific type of tear, 3–5 cm in size, were included. Therefore, these results cannot be generalized for all rotator cuff tears. The reason for selecting large tears was that single-row and double-row repair results are the same in small tears.

## 5. Conclusions

Recent studies have shown that transosseous-equivalent repairs provide the best strength and pressure in the footprint. Using fewer implants when using a transosseous-like technique may be due to practices in different centers restricting treatment costs. This study evaluated the functional results and re-rupture rates of two techniques, one applying one anchor and one push lock, and the other applying two anchors and two push locks. The study showed that the functional results were similar, and although the re-rupture rate was higher in Group 1, it was not statistically significant. In light of these results, the first technique seems preferable, as costs are lower because fewer implants are used, and the functional results are almost the same for surgeons at the start of the learning curve. Despite the disadvantages of the second technique, such as the higher cost, longer operating time, and frequency of intraoperative implant complications, it provides a more stable and stronger fixation.

## Figures and Tables

**Table 1:** Demographic and clinical characteristics of the study groups.

	Group 1	Group 2
Number of Patients	26	27
Female (n, %)	18 (69.2%)	22 (81.4%)
Male (n, %)	8 (30.8%)	5 (18.6%)
Mean Age (years)	59.07	62
Dominant Side Affected (n)	19	17
Bankart Repair (n)	2	2
SLAP Repair (n)	2	1
Biceps Tenodesis (n)	-	1
Biceps Tenotomy (n)	-	1
Subscapularis Repair (n)	-	1
Acromioplasty (n)	26	27

**Table 2:** Comparisons of the two patient groups’ preoperative and postoperative shoulder abduction angles.

**Preoperative Shoulder Abduction Degree**		**Group 1**	**Group 2**	**Total**
0–30	n %	1 3.8%	0 0%	1 1.9%
31–60	n %	6 23.1%	2 7.4%	8 15.1%
61–90	n %	10 38.5%	8 29.6%	18 34%
91–120	n %	5 19.2%	9 33.3%	14 26.4%
121–150	n %	1 3.8%	6 22.2%	7 13.2%
151–180	n %	3 11.5%	2 7.4%	5 9.4%
Total	n %	26 100%	27 100%	53 100%
**Postoperative Shoulder Abduction Degree**		**Group 1**	**Group 2**	**Total**
61–90	n %	2 7.7%	1 3.7%	3 5.7%
91–120	n %	3 11.5%	4 14.8%	7 13.2%
121–150	n %	6 23.1%	3 11.1%	9 17%
151–180	n %	15 57.7%	19 70.4%	34 64.2%
Total	n %	26 100%	27 100%	53 100%

**Table 3:** Comparisons of the preoperative and postoperative Constant scores between the two groups.

Group		Preop Constant	Postop Constant
1	Mean	41.73	84.1
	Median	42	88.5
	N	26	26
	Std. Deviation	10.05	15.8
	Minimum	24	41
	Maximum	68	100
2	Mean	42.85	84
	Median	44	88
	N	27	27
	Std. Deviation	9.5	15.45
	Minimum	27	39
	Maximum	68	100
Total	Mean	42.3	84.04
	Median	42	88
	N	53	53
	Std. Deviation	9.71	15.5
	Minimum	24	39
	Maximum	68	100

**Table 4:** Grouping of the Constant scores as excellent, good, moderate, and poor.

Postoperative Constant Score		Group 1	Group 2	Total
Poor	n %	6 23.1%	5 18.5%	11 20.8%
Moderate	n %	1 3.8%	1 3.7%	2 3.8%
Good	n %	6 23.1%	10 37%	16 30.2%
Excellent	n %	13 50%	11 40.7%	24 45.3%
Total	n %	26 100%	27 100%	53 100%

## Data Availability

All data can be transmitted to the journal if requested.
